# Artificial intelligence for venous thromboembolism risk stratification in surgical patients: a systematic review

**DOI:** 10.1007/s11239-026-03257-9

**Published:** 2026-03-06

**Authors:** Kavin Shah, Michael Gadelrab, Emily A. Brennan, Maggie L. Westfal, Colleen A. Donahue, John Del Gaizo, Arman Kilic, Thomas Curran

**Affiliations:** 1 College of Medicine, Medical University of South Carolina, Charleston, SC USA; 2 MLIS, MUSC Libraries, Medical University of South Carolina, Charleston, USA; 3 Department of Surgery, Medical University of South Carolina, Charleston, USA

**Keywords:** Artificial intelligence, Machine learning, Venous thromboembolism, Surgical patients, Risk stratification, Systematic review

## Abstract

Venous thromboembolism (VTE), including deep vein thrombosis (DVT) and pulmonary embolism (PE), is a significant complication in surgical patients. Artificial intelligence (AI) and machine learning (ML) may enhance VTE risk stratification by leveraging novel parameters in electronic medical records (EMRs). We aim to systematically review the role of AI and ML in stratifying the risk of postoperative VTE in surgical patients. A systematic literature search was conducted in PubMed, Scopus, and CINAHL Complete through May 24, 2024, and registered in PROSPERO (CRD420250522393). Eligible studies included primary research on postoperative adult patients undergoing any surgery that reported VTE risk assessment using the area under the receiver operating characteristic curve (AUC). Risk of bias was assessed, and the Wilcoxon signed-rank test was used to compare AUCs. 34 studies met the inclusion criteria, of which 22 directly compared AI models with non-AI models and were used for analysis. AI models showed higher discrimination than non-AI comparators within the same cohorts, with a median ΔAUC of + 0.10 (IQR 0.03–0.21; Wilcoxon signed-rank test, *n* = 22, *p* < 0.001). 31 studies were found to have a high risk of bias in model development, with only 12 reporting calibration metrics. Studies were limited to the United States (17) and China (17), with substantial surgical heterogeneity. Across head-to-head studies, AI models consistently showed higher discrimination than their non-AI comparators within the same cohorts. Future research should prioritize external validation, standardized reporting of calibration metrics, and generalizability.

**Graphical abstract:**

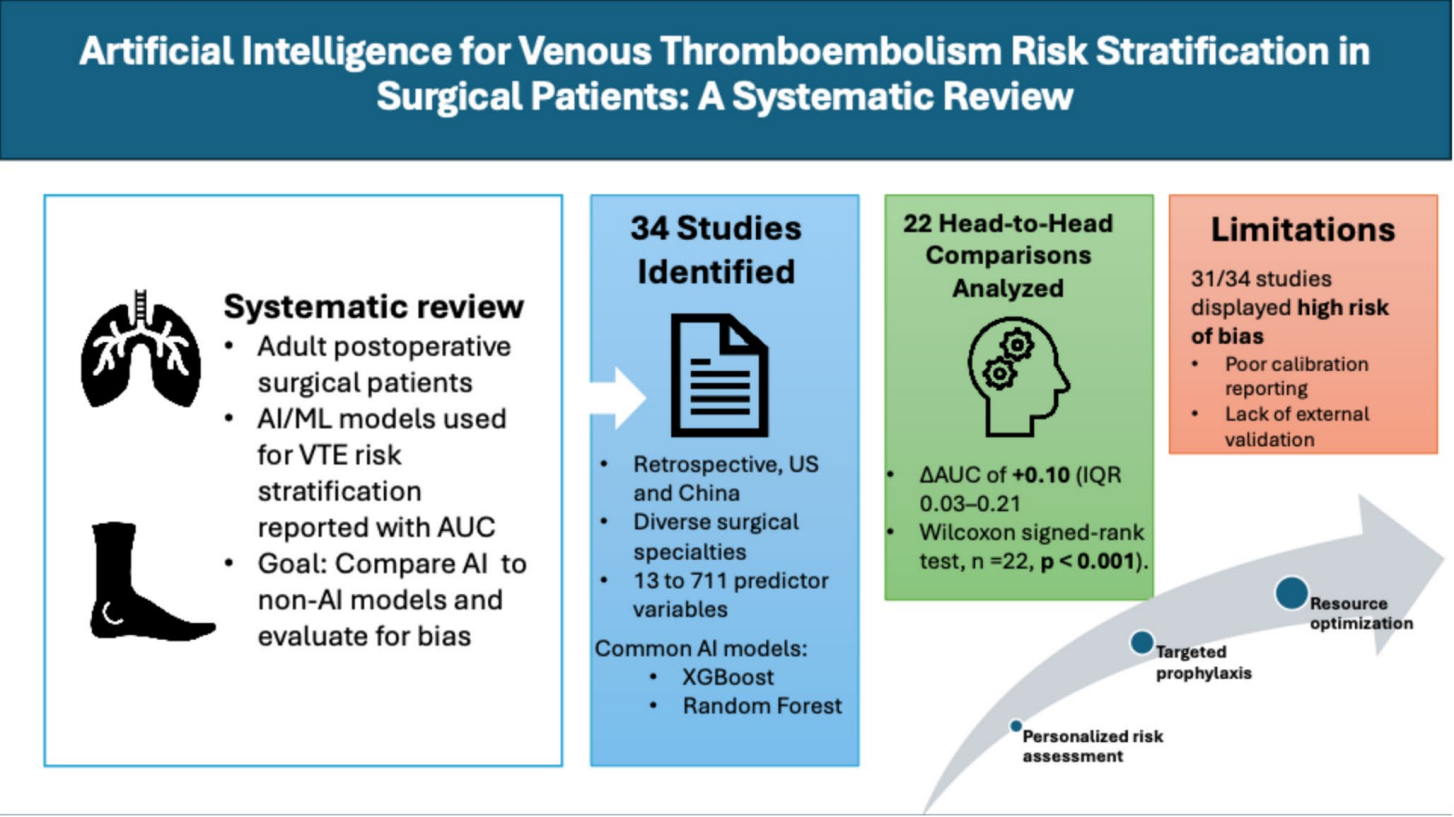

**Supplementary Information:**

The online version contains supplementary material available at 10.1007/s11239-026-03257-9.

## Introduction

Venous Thromboembolism (VTE), including deep vein thrombosis (DVT) and pulmonary embolism (PE), is a significant postoperative complication in surgical patients. Deaths related to VTE in the United States are estimated to be 296,370 annually, with a third of these presenting as sudden fatal PE and only 21,223 (7%) of these patients having been accurately diagnosed and treated prior [1]. Among surgical patients, evaluation of a national data set revealed that symptomatic VTE was associated with a 4-fold increase in 30-day mortality (16.9% vs. 4.4%; *p* < 0.001) [2]. Additionally, one study evaluating over 1.6 million surgical cases across 76 procedures estimated the 90-day VTE incidence to be 0.8%, with 56% of these events occurring after hospital discharge [3]. These events incur substantial financial costs, with direct medical expenses ranging from $12,000 to $15,000 among first-year survivors and annual healthcare system costs of approximately $7 billion to $10 billion [4].

Despite the high morbidity and cost associated with VTE, risk stratification in patients undergoing surgery is limited. The Caprini model is the most frequently utilized tool for VTE risk stratification, having been validated in 4.2 million patients across 22 studies [5]. However, a score of 5 on the Caprini Model, associated with “high risk”, was found to correspond to a highly variable VTE risk across surgical fields, ranging from 1.6% in vascular and general surgery patients to 10.5% in thoracic surgery patients [5]. A previous systematic review of 8 studies assessing the performance of the Caprini model in surgical patients reported C-statistics ranging from 0.53 to 0.87, suggesting weak performance across several studies [6]. Furthermore, procedure- and diagnosis-specific models utilizing national data, such as those for colectomies performed for colorectal cancer, also showed only modest performance (C-statistic 0.65) [7]. The variable accuracy of the Caprini score suggests a need for improved VTE risk stratification in surgical patients.

Virchow’s triad of venous stasis, hypercoagulability, and endothelial injury serves as a distillation of risk factors for VTE [8]. Given that the revised Caprini Model uses only 35 discrete variables, there are parameters in electronic medical records (EMRs) that relate more closely to these underlying mechanisms, especially in surgical patients. Artificial Intelligence (AI) and Machine Learning (ML) have been shown to improve VTE risk prediction and diagnosis using EMR data [9]. A systematic review of 20 studies across various cohorts found that AI demonstrated improved VTE prediction capabilities compared to non-AI risk models [10]. However, a systematic review focused on the role of AI/ML in VTE risk stratification in surgical patients has not been performed.

We hypothesize that AI and ML can leverage novel parameters within the EMR to improve VTE risk stratification in surgical patients. This study aims to systematically review the performance of AI and ML models for stratifying VTE risk in surgical patients and compare their performance with that of conventional methodologies.

## Methods

This study was conducted in accordance with the Preferred Reporting Items for Systematic Reviews and Meta-Analyses (PRISMA) guidelines and was registered in PROSPERO (CRD420250522393).

### Data sources and search strategies

A librarian conducted a systematic literature search in PubMed (U.S. National Library of Medicine, National Institutes of Health), Scopus (Elsevier), and CINAHL Complete (EBSCOhost). The databases were searched from inception through May 24, 2024. The search strategies employed a combination of subject headings (e.g., MeSH in PubMed) and keywords related to AI/ML, risk stratification/prediction, and perioperative venous thromboembolism. The PubMed search strategy was modified for the other two databases, replacing MeSH terms with appropriate subject headings when available and retaining similar keywords. English language filters were applied. A second librarian peer-reviewed the database search strategies using a modified PRESS peer review form [11]. The search strategies for each database are detailed in Appendix 1.

### Study selection

References were exported into the review management software, Covidence [12], for de-duplication and study selection. Eligible studies included primary research articles that evaluated the use of AI/ML to predict or diagnose VTE in perioperative patients aged 18 years or older undergoing any surgery. Studies reporting VTE risk assessment using the area under the receiver operating curve (AUC) were selected. Studies evaluating the use of AI/ML to diagnose VTE from imaging were excluded. Two reviewers [KS, MG] independently screened titles and abstracts to determine eligibility. Conflicts were resolved through consensus review. Following the same process, two reviewers [KS, MG] then independently screened full-text articles. Studies sharing the same first author surname and year of publication were distinguished using numerical suffixes (1) and (2) to avoid ambiguity when referencing distinct articles.

### Data extraction

Data extraction was performed similarly to prior studies [10], using the CHARMS checklist [13], and was conducted independently by two authors [KS, MG]. Details extracted include author, year of publication, indication for surgery, characteristics of surgery, inclusion and exclusion criteria, outcome incidence, AI models, internal validation, discrimination and calibration, and AUC/C-stat. Studies lacking calibration data were presumed not to have performed calibration.

### Risk of bias assessment

Risk of Bias quality assessment was conducted independently by two authors [KS, MG] using the PROBAST (Prediction model Risk of Bias Assessment Tool) [14, 15]. Domains evaluated for bias and applicability regarding the models include predictors, population, outcome, and analysis. Studies were classified as having a high, low, or unclear level of bias. Throughout the data selection, extraction, and assessment process, any discrepancies were resolved through consensus review mediated by author TC.

### Statistical analysis

Given the heterogeneity of patient populations, data sources, AI models, and surgeries, a meta-analysis was deferred in favor of a systematic review [16]. Subgroup and sensitivity analyses were not performed because substantial heterogeneity across surgical populations, AI models, and study designs yielded small, clinically incomparable subgroups, thereby limiting statistical power and interpretability. Quantitative comparisons were restricted to the 22 studies that evaluated both an AI model and a non-AI comparator within the same patient cohort. For each study, the difference in discrimination was calculated as the paired difference in AUC (ΔAUC = AUC_AI − AUC_non-AI). Because the number of paired comparisons was limited and the distribution of ΔAUC values could not be assumed to be normal, a non-parametric Wilcoxon signed-rank test was used to assess whether the median ΔAUC differed from zero. All tests were two-sided. For studies reporting multiple AI and non-AI models, the highest AUC in each category was selected for reporting and analysis.

## Results

### Study identification

**Fig. 1:**
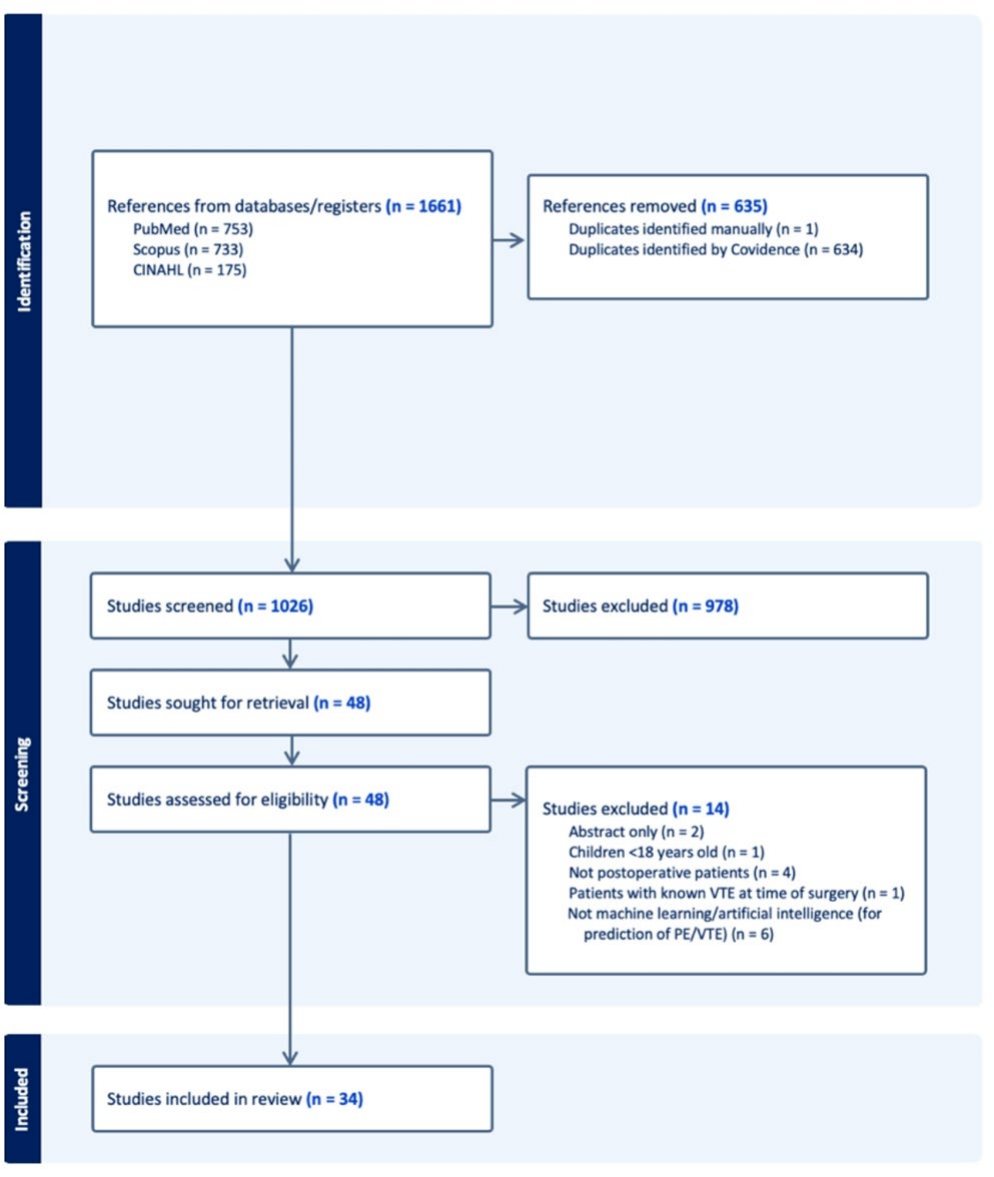
PRISMA Flow Diagram. Flow Diagram illustrating the systematic review process. A total of 1,661 records were identified across PubMed, Scopus, and CINAHL. After duplicate removal and screening, 34 studies met the inclusion criteria for final analysis. Reasons for exclusion during full-text review are detailed

The resulting articles were retrieved, screened, excluded, and extracted, and are presented in the PRISMA flow diagram (Fig. 1). Database searches identified 1,661 studies. 635 duplicates were removed. Title and abstracts of the remaining 1,026 eligible studies were screened, 978 of which were deemed irrelevant because they did not meet the eligibility criteria. A full-text review of the remaining 48 studies resulted in the exclusion of 14 studies for failing to meet the inclusion criteria. 34 studies were included in this systematic review, of which 22 conducted head-to-head comparisons with non-AI models. Interest in this topic has increased over time, with 11 included studies originating between 2018 and 2021 and 23 included studies from 2022 to 2024.

### Study and patient characteristics of included studies

The characteristics of the 22 studies used for analysis are presented in Tables 1 and 2, with details for all 34 studies included in Tables 3 and 4 in the Appendix. The studies lacking Non-AI comparators were 22, 23, 24, 30,34, 36, 38, 40, 42, 49,50, and 54. All 34 included studies were prediction models for risk stratification of postoperative VTE. Among these, five studies [17–21] specifically reported DVT outcomes, while two studies [22, 23] reported outcomes for DVT and PE individually. The studies were conducted in the United States (17) and China (17) and published between 2018 and 2024. All were retrospective in design and had patient populations ranging from 100 to 662,772. The populations consisted of post-operative patients, with significant heterogeneity across studies in the surgeries performed. Two studies [24, 25], separately evaluated data for general and orthopedic surgery. Among all studies, data were primarily sourced from medical databases and EMRs. 22 studies explicitly stated their method for determining VTE incidence.

**Table 1:** Characteristics of analysed Studies

Study ID	Country	Indication for Intervention	Total, N	Outcomes	VTE Incidence	DVT Incidence	PE Incidence	Mean Follow up Time
Arvind [39]	USA	Cervical spine disorders requiring anterior cervical discectomy and fusion (ACDF)	20,879	VTE	0.30%	NR	NR	NR
Chen [19] (1)	China	Patients with abdominal related emergency general surgery	10,993	VTE	5.60%	NR	NR	NR
Chen [41] (2)	China	Accessory surgery, total hysterectomy, uterine fibroid, laparoscopic gynecological malignant tumor surgery	489	DVT	NR	8.38%	NR	NR
Ding [27]	China	Hip disease, unspecified	1481	VTE	5.13%	NR	NR	Inpatient
Gowd [43]	USA	Osteoarthritis, rheumatoid arthritis, posttraumatic/instability arthropathy, avascular necrosis, and fracture	17,119	DVT, PE	NR	0.34%	0.32%	< 30 days from Discharge
He [35]	China	Trauma	903	VTE	21.40%	NR	NR	Inpatient
Kim [37] (1)	USA	Adult scoliosis (progression of childhood scoliosis), degenerative scoliosis, sagittal and coronal imbalance, and iatrogenic deformity (with or without spinal stenosis)	5,818	VTE	2%	NR	NR	NR
Kim [52] (2)	USA	Various degenerative conditions of the lumbar spine	22,629	VTE	0.98%	NR	NR	Other: <30 days post-op
Lin [53]	China	Cervical Cancer Uterine Cancer Ovarian Cancer	1087	VTE	6.00%	NR	NR	< 30 days post-op
Lopez [51]	USA	ACL tear	21,636	DVT	NR	0.50%	NR	< 30 days from Discharge
Ma [25]	China	NR	9213	VTE	12.60%	NR	NR	
Nudel [32]	USA	Obesity and associated metabolic diseases	436,807	VTE	0.46%	NR	NR	< 30 days from Discharge
Qiao [44]	China	Sellar region tumors (including pituitary adenoma, craniopharyngioma, tuberculum sellae meningioma, chordoma, and cystic lesions)	3818	VTE	3.20%	NR	NR	31–90 days from Discharge
Qin [45]	China	Colorectal Carcinoma	1,191	VTE	10.80%	NR	NR	< 30 days post-op
RasouliDezfouli [46]	USA	Hip or Knee replacement	541,354	VTE	0.75%	NR	NR	Other: <30 days post-op
Ren [47]	USA	NR	74,417	VTE	5.60%	NR	NR	NR
Wang [49]	China	NR	81,505	VTE	0.69%	NR	NR	NR
Wang [48] (1)	USA	Degenerative spinal pathology	13,500	VTE	0.95%	NR	NR	< 30 days from Discharge
Wang [21] (2)	China	NR	6,897	DVT	NR	16.80%	NR	Inpatient
Wei [17]	China	Lower limb fracture	4,424	DVT	NR	4.68%	NR	NR
Yan [31]	China	Inguinal Hernia	1600	VTE	1%	NR	NR	Inpatient
Zhang [18]	China	Gastrointestinal Tumors	845	DVT	NR	18%	NR	Other: 14 days post-op

VTE = venous thromboembolism; DVT = deep vein thrombosis; PE = pulmonary embolism; EMR = electronic medical record, NR = Not reported

**Table 2:** Characteristics of analysed models

Study ID	AI Models	Data Input	Variable Collection Timing	Training Cohort	Testing Cohort	Internal Validation method	External Validation method	Top Performing AI model	AUC - AI Model	Top Performing Non-AI Model	AUC - Non-AI Model	Discrimination Method	Calibration Method
Arvind [39]	RF; ANN; LR; SVM	6 Variables	Preoperative	14,615	6,264	5-fold-cross validation	NR	ANN	0.656	ASA physical status classification	0.397	AUC	NR
Chen [19] (1)	RF; LR; SVM; XGBoost; Naive Bayes	33 Variables	Preoperative	7695	3298	10-fold cross validation	NR	Random Forest	0.8403	ASA	0.7131	Area Under the Curve, F1 score, and sensitivity	NR
Chen [41] (2)	RF; ANN; Generalized Linear Regression	35 clinical indicators	Preoperative	342	147	Cross Validation	NR	Random Forest	0.862	Generalized Linear Regression Model (GLRM)	0.709	AUC	Calibration curves
Ding [27]	LR; XGBoost; k Nearest Neighbor (KNN); Multilayer Perceptron Adaptive boosting Gradient boosting tree model	67 pre-operate clinical characteristics	Preoperative and Postoperative	70%	30%	10-fold cross validation	NR	XGBoost	0.982	PLOS	0.716	AUC, sensitivity, specificity	NR
Gowd [43]	RF; LR; k Nearest Neighbor (KNN); DT; Naive-Bayes Gradient boosting trees	22 Variables	Preoperative	13,697	3,422	Cross-validation	NR	Random Forest	0.58	ASA classification	0.6	AUC	NR
He [35]	RF; LR; LASSO; Ridge Regression ElasticNet Regression Mutual Information Entropy	69 Variables	Preoperative, Intraoperative, and Postoperative	NR	NR	10-fold validation	NR	LR + RF + Caprini	0.8	Caprini Risk Assessment Model	0.773	AUC, TPR, FPR, accuracy, and precision	NR
Kim [37] (1)	ANN; LR	12 Variables	Preoperative	4,073 (70%)	1,746 (30%)	5-fold cross-validation	NR	ANN	0.542	LR	0.547	AUC	NR
Kim [52] (2)	ANN; LR	12 Variables	Preoperative	70%	30%	Split of data	NR	ANN	0.567	LR	0.588	AUC	NR
Lin [53]	LR; DT	19 Variables	Preoperative	870	217	10-fold cross validation	NR	Decision Tree	0.95	Logistic Regression	0.722	AUC	NR
Lopez [51]	ANN; LR	14 Variables	Preoperative	17,309	4,327	Split of Data (random)	None	ANN	0.804	Logistic Regression	0.608	AUC	Calibration Curves
Ma [25]	RF; LR; XGBoost; Multi-Layer Perceptron TSGB (o, b,k)	Features from electronic health records (EHR), Specific number not stated.	Preoperative, Intraoperative, and Postoperative	70%	30%	Split of Data (Random)	NR	O: TSGB(k) variant S: TSGB(o) variant	O: 0.7639, S: 0.8062	Logistic Regression	O: 0.6894, S: 0.7693	AUC	NR
Nudel [32]	ANN; LR; XGBoost	All preoperative clinical Variables available in MBSAQIP database	Preoperative	218,403	109,202	Split of Data (Random)	None	XGBoost	0.67	LR	0.64	AUC	NR
Qiao [44]	RF; ANN; LR; SVM; LASSO; Ensemble algorithm, linear discriminant analysis Gradient Boost Machine	25 Variables	Preoperative and Postoperative	2772	1046	5-fold cross validation	NR	Linear Discriminant Analysis	0.869 in training, 0.884 in validation	D-Dimer	0.812	AUC	Calibration Intercept, Calibration Slope and Brier Score
Qin [45]	RF; LR; SVM; XGBoost; Multilayer Perceptron (MLP), Long Short-Term Memory (LSTM) Network	Patient level factors, cancer level factors, treatment level factors, and laboratory data	Preoperative and Postoperative	30%	1,191	10-fold cross validation, with total dataset validation	NR	XGBoost	0.908	Caprini	0.769	AUC	Brier Score.
RasouliDezfouli [46]	RF, Tree Enemble (TE), Gradient Boosted Trees (GBT), Fully connected deep neural network (FCDNN)	100 Variables	Preoperative	70%	30%	Holdout validation with random stratified sampling.	NR	Fully Connected Deep Neural Network (FCDNN)	0.901	Traditional Risk Scoring Table	0.639	AUC	NR
Ren [47]	RF; Generalized Additive Models (GAMs)	135 Variables	Preoperative, Intraoperative, and Postoperative	52,117	22,300	5-fold cross-validation on development cohort, bootstrapping, split of data (random)	None	Random Forest	0.82	Surgeon Assessment	0.6	AUC	NR
Wang [49]	RF; LR	Risk factors from Caprini RAM, and additional risk factors from previous works.	Preoperative	53,410	14,742	Ten-fold cross validation	NR	Random Forest	0.839	Caprini	0.758	AUC	NR
Wang [48] (1)	LR; XGBoost	16 Variables	Preoperative	80%	20%	Split of Data (Random)	No external validation performed.	XGBoost	0.716	Charlson (CCI), LR	CCI: 0.515 LR: 0.709	AUC	NR
Wang [21] (2)	RF; LR; SVM; XGBoost; Ensemble Backpropagation Neural Network	5 categories of Variables, specific number not reported	Preoperative	80%	20%	5-fold cross validation		Ensemble Model	0.9206	Caprini score	0.5703	AUC	NR
Wei [17]	RF; LR; SVM; XGBoost; Multilayer Perception Model	36 Variables	Preoperative	NR	NR	5-fold cross-validation with hyperparameter tuning.	NR	XGBoost	0.979	Logistic Regression	0.821	AUC	NR
Yan [31]	RF; LR; XGBoost; SVM_Linear, SVM_RBF, LightGBM, TabNet, AdaBoost, CatBoost	26 Variables	Preoperative, Intraoperative, and Postoperative	1,280	320	Split of Data (Random)	NR	TabNet	0.74	Logistic Regression	0.67	AUC	NR
Zhang [18]	RF; LR; SVM; XGBoost; LASSO; DT	23 Variables	Preoperative	586	259	Split of Data (Random)	NR	XGBoost	1	Logistic Regression	0.992	AUC	Calibration curves, decision curve analysis (DCA), and precision-recall curves (PRC).

AUC = area under the curve; RF = Random Forest; LR = logistic regression; SVM = support vector machine; ANN = artificial neural network; DT = Decision Tree AUC values reflect model discrimination; calibration metrics were reported in 12 of 34 studies

### Models of AI

Many studies utilized multiple AI algorithms in their evaluation. The AI models with the highest AUC across the studies varied significantly. Of the included studies, XGBoost was the overall top-performing AI model, ranking first in 9 of 34 studies (26.5%), with an average C-statistic of 0.865. It was followed by Random Forest, which was best in 7 studies, with an average C-statistic of 0.796. The training and testing cohort sizes were reported in 29 studies, and predictor variables initially included in the models ranged from 13 to 711, with 6 studies not reporting a specific number.

### Performance measures

**Fig. 2:**
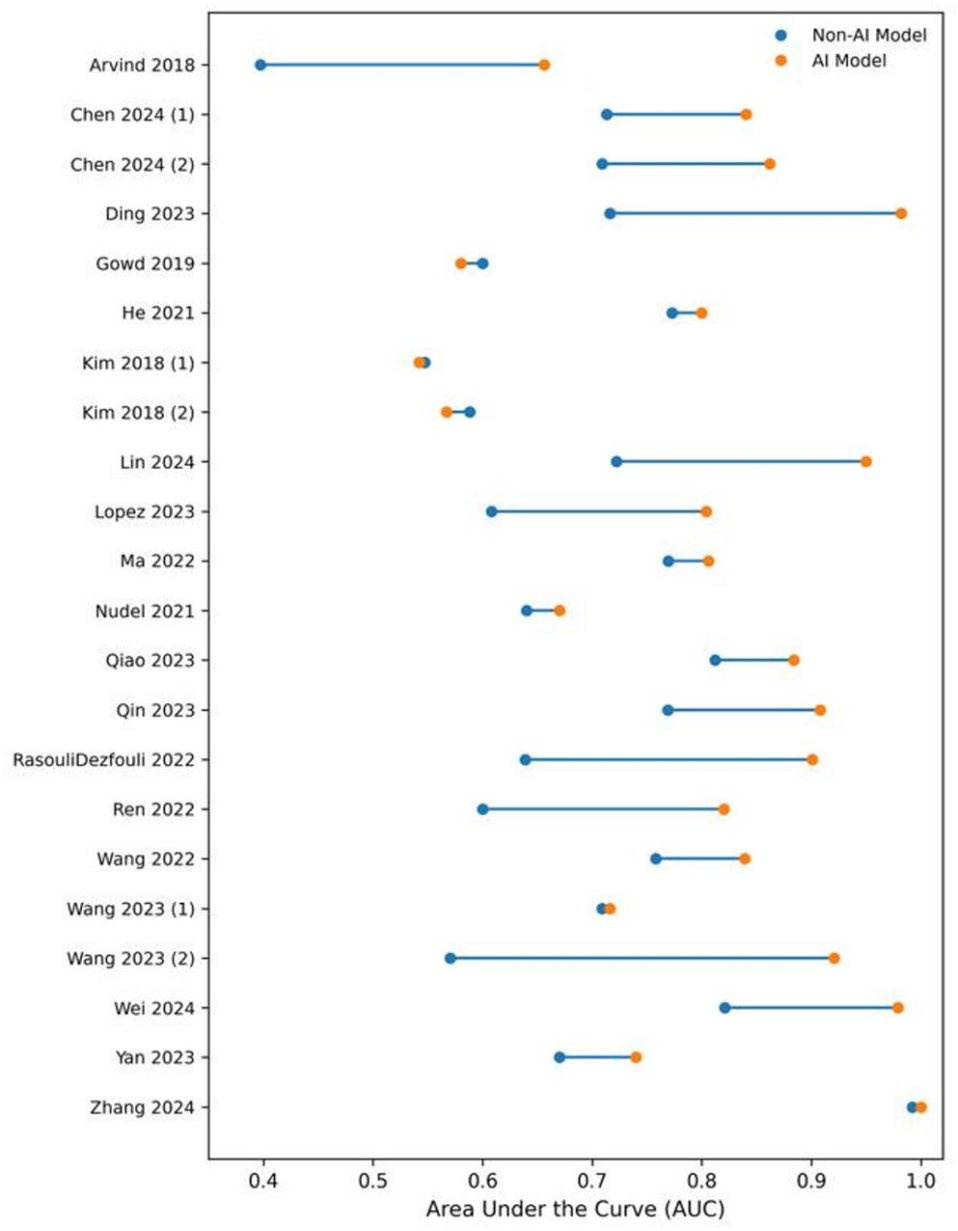
Dumbbell Plot. Plot highlighting the ΔAUC of the 22 studies utilized for analysis. Each horizontal line represents a study that evaluated both an AI-based model and a non-AI comparator within the same patient cohort. Circles indicate the area under the receiver operating characteristic curve (AUC) for the top-performing AI model and the corresponding non-AI model, with line length reflecting the difference in discrimination (ΔAUC)

The performance of the AI models was evaluated using Discrimination and Calibration, as seen in Table 1. Discrimination was reported as the AUC and was present in all studies. Of the 22 studies analyzed, AI-based models demonstrated overall higher discrimination than non-AI models, with a median ΔAUC of + 0.10 (IQR 0.03–0.21; Wilcoxon signed-rank test, *p* < 0.001). The resulting ΔAUC for each study is displayed in Fig. 2. Calibration was reported in only 12 studies, limiting the real-world applicability of these models.

### Risk of bias and applicability assessment

**Fig. 3:**
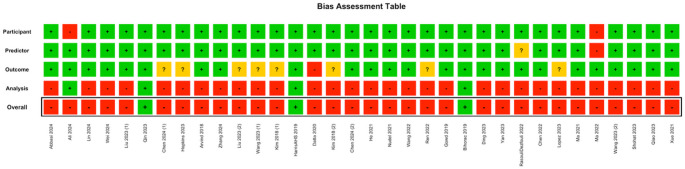
Risk of Bias Assessment. Summary heatmap showing the risk of bias using the PROBAST tool. Green (+) indicates low risk, yellow (?) indicates unclear risk, and red (-) indicates high risk

A summary of the risk of bias and applicability of the AI models is presented in Fig. 3 and Appendix 4. Most studies had a high risk of bias as determined using the PROBAST, with the primary source being inadequate AI model calibration. Almost all AI models exhibited a low level of concern regarding applicability in the context within which they were trained.

## Discussion

We identified 34 studies evaluating the use of AI models to stratify VTE risk in postoperative surgical patients. Interest in this topic increased over the study period, with two-thirds of the included studies published in the last three years. Twenty-two of the included studies compared AI models to non-AI models; however, these comparators were predominantly internally developed statistical models, such as logistic regression, with only three direct comparisons to validated risk assessment models. Our study displayed that, of the 22 head-to-head comparisons within the patient cohorts, AI-based models demonstrated consistently higher discrimination than non-AI models, as reflected by a positive median difference in AUC (Median ΔAUC of + 0.10, (IQR 0.03–0.21)) and a statistically significant Wilcoxon signed-rank test (*n* = 22, *p* < 0.001). A paired, nonparametric statistical approach was necessary given the bounded nature of AUC values, the limited number of head-to-head comparisons, and the heterogeneity of the included studies. By focusing on within-study differences and evaluating median effects, the Wilcoxon signed-rank test provides a conservative assessment of discrimination performance without assuming normality or cross-study comparability.

While AI models demonstrated improved discrimination, certain non-AI models also achieved strong predictive performance, notably the Logistic Regression model in Zhang et al. [18], with an AUC of 0.992, suggesting that well-designed non-AI tools may remain valuable in particular clinical contexts. A common theme throughout the evaluated studies was that AI-informed VTE risk stratification may improve predictive accuracy and enable more personalized perioperative VTE prophylaxis for high-risk patients. Although this review focused on surgical patients, prior systematic reviews have examined AI and ML applications for VTE risk prediction in non-surgical and general hospitalized populations. Wang et al. [26] conducted a meta-analysis of 12 studies across diverse patient cohorts, finding a pooled AUC of 0.98 for AI models. Similarly, Chiasakul et al. performed a systematic review and pooled analysis of 20 studies in hospitalized patients, finding that AI models consistently outperformed conventional risk assessment tools for VTE prediction. The mean AUC for AI versus conventional methods was 0.79 (95% CI: 0.74–0.85) versus 0.61 (95% CI: 0.54–0.68), respectively (*p* < 0.001) [10]. Our findings align with the existing literature and reinforce the expanding utility of AI and ML for VTE risk stratification across diverse patient populations in both surgical and non-surgical settings.

While our findings indicate that AI-based approaches outperform non-AI comparators within the same cohort, they must be evaluated in the context of several limitations. In this review, logistic regression models were categorized as non-AI statistical comparators. While logistic regression can be broadly considered a supervised learning method from a computational perspective, it is not an established clinical risk assessment tool designed for bedside use. Direct comparisons between AI-based models and validated clinical risk scores, including the Caprini score, were unavailable in most included studies. This substantially limits conclusions about the clinical superiority of AI-based models over established bedside risk assessment tools, which are specifically designed to guide perioperative VTE risk stratification and thromboprophylaxis decision-making. Furthermore, most logistic regression models used as non-AI comparators were newly developed and internally evaluated within their respective studies rather than representing externally validated clinical prediction models. As such, observed performance differences should be interpreted as study-specific benchmarking against internally derived statistical models rather than definitive evidence of superiority over validated clinical risk assessment instruments.

Although AI models demonstrated improved discrimination compared with non-AI models in the evaluated studies, judging clinical readiness based on discrimination alone is insufficient, as superior discrimination does not guarantee accurate risk estimation. Many studies emphasized discrimination metrics, such as AUC, without consistently reporting sensitivity and specificity at clinically meaningful thresholds, thereby limiting insight into trade-offs between false negatives and false positives. Because prophylaxis decisions require balancing missed VTE events against bleeding risk, the absence of threshold-based performance metrics constrains the clinical interpretability of these models.

Of the 34 included studies, only 12 (35.3%) reported calibration metrics, further limiting real-world applicability. Calibration contextualizes discrimination by assessing whether predicted probabilities correspond to observed event rates and can be meaningfully applied to threshold-based clinical decision-making. These models must not only identify higher-risk patients but also assign accurate absolute risk estimates. Without calibration, a model may correctly rank patients by relative risk while systematically over- or underestimating absolute risk, potentially leading to inappropriate prophylaxis decisions.

In addition, the absence of external validation in many AI models is concerning, as it increases the risk of overfitting and limits generalizability. Accordingly, the near-perfect discrimination reported by models such as those by Zhang et al. [18] (AUC = 1.000), Ding et al. [27] (AUC = 0.982), and Wei et al. [17] (AUC = 0.979) should be interpreted with caution. Findings of near-perfect discrimination in retrospective clinical datasets may reflect overfitting, data leakage, or limited external validity rather than true out-of-sample performance. The lack of prospective validation further exacerbates these limitations, as model performance may differ when applied to prospectively collected data and real-world clinical workflows.

31 of the 34 studies (91%) demonstrated high bias in model development, with all but one failing in the Analysis criteria of the PROBAST. The studies primarily failed in this domain due to inadequate reporting of calibration metrics, as previously mentioned, or to internal validation that consisted solely of a random data split. Few studies evaluated how model outputs would be integrated into perioperative workflows or assessed downstream clinical impact. In particular, the implications of AI-based risk stratification for thromboprophylaxis decision-making remain unclear, as most models were not linked to actionable treatment plans, changes in prophylaxis intensity, or patient-centered outcomes such as VTE incidence or bleeding risk. Thus, the clinical relevance of improved discrimination remains uncertain in the absence of evidence demonstrating meaningful influence on perioperative management. Although the models rated well on applicability, as seen in Appendix 4 must be cautious about early clinical adoption, as applicability in this context is limited to the cohort on which the models were trained and tested. This once again stresses the need for improved calibration and external validation reporting to increase transparency of these models.

The included studies demonstrated substantial heterogeneity, particularly in terms of surgical specialties and procedures. Our analysis captured a diverse range of surgical procedures, such as cardiac operations requiring cardiopulmonary bypass, orthopedic interventions (lower limb fractures, hip/knee arthroplasty), gastrointestinal surgeries (colorectal carcinoma, gastric cancer), neurosurgical cases (sellar region tumors), gynecological procedures (hysterectomy for various malignancies), and general surgical cases (inguinal hernia repair). Eight studies failed to specify precise surgical types, while several others covered multiple distinct procedures. This wide variation in surgical contexts presents significant challenges when interpreting findings, as baseline VTE risk profiles differ substantially between lower-risk ambulatory procedures and high-risk oncological surgeries. The pathophysiological mechanisms driving VTE risk likely vary by surgical approach, with different relative contributions from Virchow’s triad components across surgical specialties. Consequently, predictors that perform well in one surgical context may be irrelevant in another, limiting our ability to draw generalizable conclusions about optimal model architectures or input variables.

This heterogeneity also complicates the practical implementation of AI models, as healthcare systems must decide whether to adopt specialty-specific models or develop a single, generalizable model applicable across diverse surgical contexts. These challenges highlight a critical consideration in AI model development: whether to pursue generalizable models applicable across surgical contexts or precise models tailored to individual procedures or patient populations. A study on postoperative VTE risk prediction across multiple surgical types found that a generalizable deep learning model performed well in multi-center validation (AUC 0.79) but had lower specificity in smaller subgroups [24]. Analysis of the included studies demonstrated that specific AI models often achieve higher AUC values and sensitivity, while generalizable models can be utilized across different surgical fields. Current risk assessment models, such as the Caprini score, fit only the generalizable model, thereby reducing their effectiveness in specific populations. Neither approach is inherently superior; generalizable models excel at broad applicability, while specific models offer greater accuracy in targeted contexts.

The identified studies were geographically restricted to the United States (17) and China (17). This raises concerns about the generalizability of the findings to healthcare systems with different patient characteristics, clinical practices, and documentation standards. Most studies relied on single-center, retrospective data from distinct patient populations or healthcare settings, without external validation. This limitation is particularly problematic for VTE prediction models, as risk factors and their relative importance may vary across different populations and surgical practice patterns. Another important limitation of these studies is the heterogeneity in follow-up duration, which compromises the comparability of VTE incidence estimates and overall model performance. A meta-analysis of 6258 studies found that the risk of VTE remains roughly 10.1% in the fourth week postoperatively, highlighting the importance of adequate follow-up duration [28]. Regarding outcomes, most studies did not differentiate between DVT and PE when developing prediction models. While both are manifestations of VTE, they have distinct risk factor profiles, clinical presentations, and disease outcomes [29] that may benefit from separate predictive approaches for optimal accuracy. This lack of distinction represents a significant limitation in the current literature, as composite VTE outcomes may mask significant differences between these two manifestations, potentially leading to suboptimal risk stratification for specific patient populations.

While these limitations warrant careful consideration, they do not diminish the role that AI models can serve in improving VTE risk stratification. Traditional VTE risk assessment tools, such as the Caprini score, rely on a standardized set of general factors that, while clinically validated [5], lack the specificity and nuance that AI models can achieve. The Caprini model assigns points based on broad categories such as age (1–5 points), BMI > 25 (1 point), major surgery (2–5 points), history of prior VTE (3 points), malignancy (2 points), and immobilization (1–2 points). These parameters, while important, treat all patients within the same broad clinical categories as similar and fail to account for the complex interactions between patient-specific physiological markers and procedural details. In contrast, AI models can detect subtle patterns by incorporating novel variables, thereby enabling more precise risk stratification. For instance, the HbA1c levels reported by Ali et al. [30] offer insight into glycemic control, while direct bilirubin levels in Ding et al. [27] provide specific information about liver function that conventional risk assessment models do not capture. AI models can also differentiate VTE risk based on specific surgical approaches, such as open versus minimally invasive [31]. In contrast, the Caprini score only accounts for “major surgery” without accounting for variations in technique. Laboratory values such as albumin [21] and hematocrit [32] were utilized as objective, quantifiable measurements of physiological status that supersede the non-AI models’ assessment of general health status. Mobility, for example, could be assessed using the AMPAC score, which may provide additional insight into functional status and offer predictive value for VTE [33]. By analyzing these detailed factors, AI models can create highly personalized risk profiles that reflect the multifactorial and patient-specific nature of VTE development, enabling more tailored prophylaxis strategies that are not currently possible with the one-size-fits-all approach of traditional scoring systems. Despite promising discrimination performance in head-to-head comparisons, the current evidence does not support premature clinical adoption of AI-based postoperative VTE risk prediction models. Most included studies relied on retrospective, single-center datasets and lacked calibration reporting and external validation across independent cohorts. Few models were evaluated across diverse surgical populations, healthcare systems, or geographic settings, limiting confidence in their generalizability. Moreover, temporal validation was rarely performed, raising concerns about model robustness to evolving clinical practices, patient demographics, and perioperative care pathways. Collectively, these limitations underscore the need for improved calibration reporting and rigorous external, temporal, and geographically diverse validation before AI-based VTE risk models can be considered for routine clinical implementation. Prospective studies comparing these models to established conventional methods and implementing them in clinical practice through real-time EMR integration will be essential to demonstrate their real-world impact on patient outcomes and resource utilization.

## Conclusion

While the evidence base remains heterogeneous, this systematic review suggests that AI and ML models can enhance VTE risk stratification in surgical patients by leveraging advanced modeling approaches and incorporating a broader set of predictive features. However, broader adoption should be contingent on rigorous calibration, external validation, and prospective evaluation to ensure reliable absolute risk estimates and generalizability across settings. Successful translation into clinical benefit will also require thoughtful implementation, including seamless EMR integration, clinician education, and ongoing performance monitoring to mitigate drift and algorithmic bias and to ensure that improved metrics translate into better prophylaxis decisions and surgical outcomes.

## Supplementary Information

Below is the link to the electronic supplementary material.

Supplementary Material 1

Supplementary Material 2

Supplementary Material 3

Supplementary Material 4

## Data Availability

All data supporting the findings of this study are available within the paper and its references.
