# Supplementary material for: Artificial intelligence for venous thromboembolism risk stratification in surgical patients: a systematic review
Source: J Thromb Thrombolysis. 2026 Mar 6;59(5):1298–312. doi: 10.1007/s11239-026-03257-9 (PMC13331843; doi:10.1007/s11239-026-03257-9)
Supplement: Supplementary file 1 — Supplementary Material 1 [file 11239_2026_3257_MOESM1_ESM.docx]

**Search Methods:**

**PubMed (U.S. National Library of Medicine, National Institutes of Health) search strategy:**

("cement emboli*"[tw] OR DVT[ti] OR "Pulmonary Embolism"[Majr] OR “pulmonary embol*”[tw] OR thromboemboli*[tw] OR thromboprophyla*[tw] OR thrombos*[tw] OR thrombotic[tw] OR “Venous Thromboembolism”[Majr] OR "Venous Thrombosis"[Majr:NoExp] OR VTE[ti]) **AND** ("Artificial Intelligence"[Mesh] OR “artificial intelligence”[tw] OR automated[ti] OR "Bayesian network*"[tw] OR “computer-aided detection”[tw] OR "deep learning"[tw] OR "Machine Learning"[Mesh] OR "machine learning"[tw] OR "natural language*"[tw] OR NLP[ti] OR "neural network*"[tw] OR "support vector machine*"[tw]) **AND** (“Algorithms”[Majr] OR algorithm*[ti] OR “automated detection”[tiab:~3] OR “automatic detection”[tiab:~3] OR detect*[ti] OR diagnos*[ti] OR “Early Diagnosis”[Majr] OR identif*[ti] OR "Logistic Models"[Mesh] OR predict*[ti] OR "prediction model*"[tw] OR "predictions model*"[tw] OR "Probability Learning"[Mesh] OR "Risk Assessment"[Mesh] OR "Risk"[Mesh] OR risk[tw] OR screen*[ti] OR surveillance[ti] OR suspected[ti]) **NOT** ("Robotics"[Majr] OR robot*[ti] OR "Meta-Analysis"[Publication Type] OR meta-analysis[ti] OR "Review"[Publication Type] OR "Systematic Review"[Publication Type] OR "systematic review"[ti])

- Filters/limits: English
- Date searched: May 24, 2024
- # of records identified: 753
- Librarian peer-reviewer: Rachel Whitney
- Date of peer-review: May 23, 2024

**Scopus (Elsevier) search strategy (basic line-by-line search):**

**#1: TITLE:** "cement emboli*" OR DVT OR “pulmonary embol*” OR thromboemboli* OR thromboprophyla* OR thrombos* OR thrombotic OR VTE

**#2: ABSTRACT:** "cement emboli*" OR “pulmonary embol*” OR thromboemboli* OR thromboprophyla* OR thrombos* OR thrombotic

**#3:** #1 OR #2

**#4: TITLE:** {artificial intelligence} OR automated OR "Bayesian network" OR “computer-aided detection” OR {deep learning} OR {machine learning} OR "natural language" OR NLP OR "neural network" OR "support vector machine"

**#5: ABSTRACT:** {artificial intelligence} OR "Bayesian network" OR “computer-aided detection” OR {deep learning} OR {machine learning} OR "natural language" OR "neural network" OR "support vector machine"

**#6:** #4 OR #5

**#7: TITLE:** algorithm* OR detect* OR diagnos* OR identif* OR {logistic models} OR predict* OR {probability learning} OR risk OR screen* OR surveillance OR suspected

**#8: ABSTRACT:** “automated detection” OR “automatic detection” OR {early diagnosis} OR {logistic models} OR "prediction model*" OR "predictions model*" OR {probability learning} OR risk

**#9:** #7 OR #8

**#10:** #3 AND #6 AND #9

**#11: TITLE:** robot* OR meta-analysis OR {systematic review}

**#12:** #10 AND NOT #11

- Filters/limits: English
- Date searched: May 24, 2024
- # of records identified: 733
- Librarian peer-reviewer: Rachel Whitney
- Date of peer-review: May 23, 2024

**Scopus (Elsevier) search strategy (advanced document search):**

( ( ( TITLE ( "cement emboli*" OR dvt OR "pulmonary embol*" OR thromboemboli* OR thromboprophyla* OR thrombos* OR thrombotic OR vte ) OR ABS ( "cement emboli*" OR "pulmonary embol*" OR thromboemboli* OR thromboprophyla* OR thrombos* OR thrombotic ) ) ) AND ( ( TITLE ( {artificial intelligence} OR automated OR "Bayesian network" OR "computer-aided detection" OR {deep learning} OR {machine learning} OR "natural language" OR nlp OR "neural network" OR "support vector machine" ) OR ABS ( {artificial intelligence} OR "Bayesian network" OR "computer-aided detection" OR {deep learning} OR {machine learning} OR "natural language" OR "neural network" OR "support vector machine" ) ) ) AND ( ( TITLE ( algorithm* OR detect* OR diagnos* OR identif* OR {logistic models} OR predict* OR {probability learning} OR risk OR screen* OR surveillance OR suspected ) OR ABS ( "automated detection" OR "automatic detection" OR {early diagnosis} OR {logistic models} OR "prediction model*" OR "predictions model*" OR {probability learning} OR risk ) ) ) ) AND NOT ( TITLE ( robot* OR meta-analysis OR {systematic review} ) ) AND ( LIMIT-TO ( LANGUAGE , "English" ) )

**CINAHL Complete (EBSCOhost) search strategy:**

("cement emboli*" OR TI DVT OR MH "Pulmonary Embolism" OR “pulmonary embol*” OR thromboemboli* OR thromboprophyla* OR thrombos* OR thrombotic OR MH "Venous Thromboembolism" OR MM "Venous Thrombosis" OR TI VTE) **AND** (MH "Artificial Intelligence+" OR “artificial intelligence” OR TI automated OR "Bayesian network*" OR “computer-aided detection” OR MH "Deep Learning" OR "deep learning" OR MH "Machine Learning+" OR "machine learning" OR MH "Natural Language Processing" OR "natural language*" OR TI NLP OR MH "Neural Networks (Computer)" OR "neural network*" OR MH "Support Vector Machine" OR "support vector machine*") **AND** (MH "Algorithms" OR TI algorithm* OR TI detect* OR TI diagnos* OR MM "Early Diagnosis" OR TI identif* OR "logistic model*" OR TI predict* OR "probability learning" OR MH "Risk Assessment" OR risk OR TI screen* OR TI surveillance OR TI suspected) **NOT** (MM "Robotics+" OR TI robot* OR TI meta-analysis OR TI "systematic review")

- Filters/limits: English
- Expanders: Apply equivalent subjects
- Narrow by Language: English
- Search modes: Boolean/Phrase
- Date searched: May 24, 2024
- # of records identified: 175
- Librarian peer-reviewer: Rachel Whitney
- Date of peer-review: May 23, 2024

**TOTAL # of records identified through database searching:** 1,661 (including 635 duplicates)

**# of records after duplicates removed:** 1,026
