# Supplementary figures and images for: Artificial intelligence for venous thromboembolism risk stratification in surgical patients: a systematic review
Source: J Thromb Thrombolysis. 2026 Mar 6;59(5):1298–312. doi: 10.1007/s11239-026-03257-9 (PMC13331843; doi:10.1007/s11239-026-03257-9)

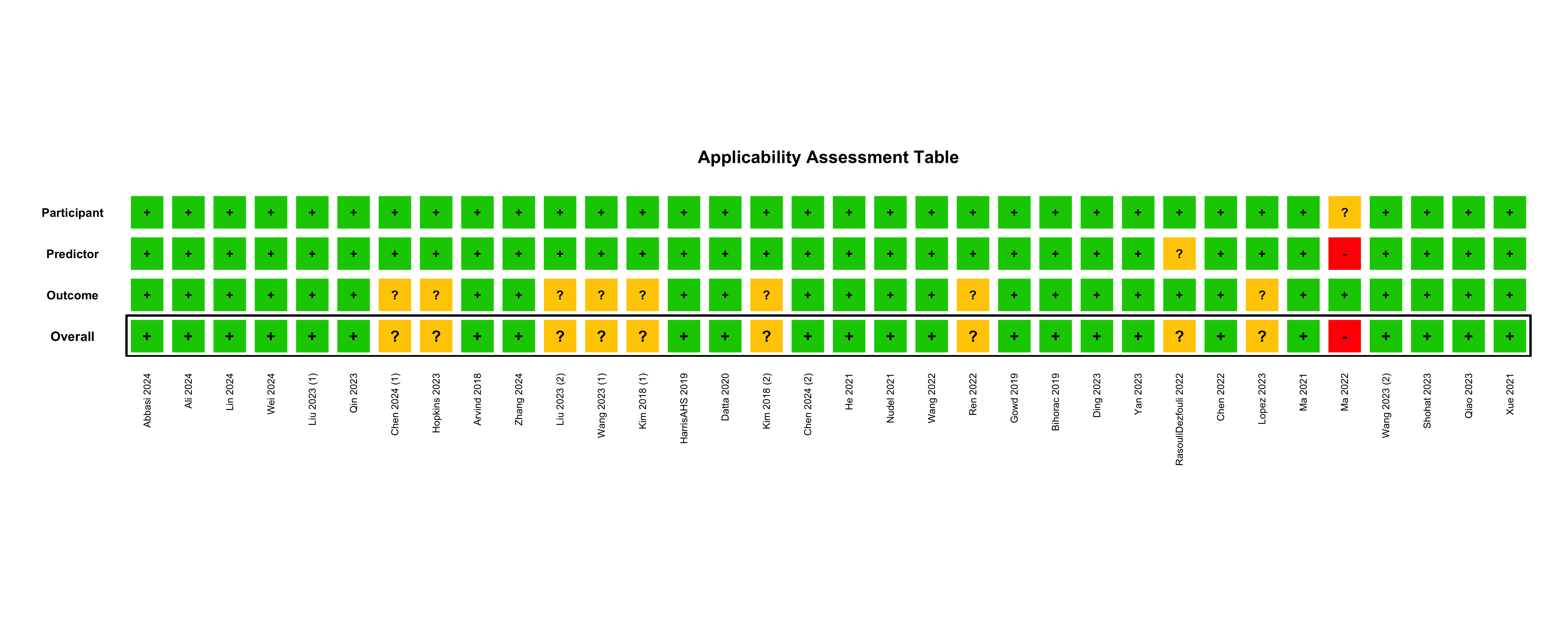

Supplement: Supplementary file 4 — Supplementary Material 4 [file 11239_2026_3257_MOESM4_ESM.png]
